# Timely Control of Gastrointestinal Eubiosis: A Strategic Pillar of Pig Health

**DOI:** 10.3390/microorganisms9020313

**Published:** 2021-02-03

**Authors:** Paolo Trevisi, Diana Luise, Federico Correa, Paolo Bosi

**Affiliations:** Department of Agricultural and Food Sciences (DISTAL), University of Bologna, 40127 Bologna, Italy; diana.luise2@unibo.it (D.L.); federico.correa2@unibo.it (F.C.); paolo.bosi@unibo.it (P.B.)

**Keywords:** weaning transition, gut microbiota, milk, antibiotic, genetics, diet

## Abstract

The pig gastrointestinal tract (GIT) is an open ecosystem in which microorganisms and their host are mutually involved and continually adapt to different factors and problems which may or may not be host dependent or due to the production system. The aim of the present review is to highlight the factors affecting the GIT microbial balance in young pigs, focusing on the pre- and post-weaning phases, to define a road map for improving pig health and the production efficiency of the food chain. Birth and weaning body weight, physiological maturation, colostrum and milk (composition and intake), genetic background, environmental stressors and management practices, antibiotic use and diet composition are considered. Overall, there is a lack of knowledge regarding the effect that some factors, including weaning age, the use of creep feed, the composition of the colostrum and milk and the use of antibiotics, may have on the gut microbiome of piglets. Furthermore, the information on the gut microbiome of piglets is mainly based on the taxonomy description, while there is a lack of knowledge regarding the functional modification of the microbiota, essential for the exploitation of microbiota potential for modulating pig physiology.

## 1. Introduction

Increases in human population have impacted the estimated demand for food of animal origin, and a rise in per capita demand has been predicted to extend from 2000 to 2030. After poultry meat (+94%) and eggs (+48%), which have shown the highest growth, pig-derived products have had an estimated growth of +44% [1,2]. However, the intensive livestock production has to face and guarantee at the same time on the one side a sufficient quantity to the demand of products of animal origin and on the other side the security for health and environment (one health approach) and more sustainable production [3,4]. Management strategies, including biosecurity and vaccination plans, as well as the genetic progress targeted to illness resistance, widely contribute to achieve these objectives [5]. However, the role of the pig physiology should not be forgotten, especially for young animals since prenatal, neonatal and post-weaning periods represent a window of intervention for modulating their physiological development and, consequently, their adaptability/resilience to the extra-uterine environment as well as their natural resistance against infectious diseases [6,7,8].

The porcine gastrointestinal tract (GIT) is an open ecosystem in which the boundaries between accepted living microorganisms and intruders need to be continuously adapted to the encounter and activity of new species, to the host dietary base and to the maturation of the host local controls. The need for a combined regulation of contiguous gastrointestinal segments, the complexity of external environments and the incidence of stressful conditions exclude the possibility of defining a static eubiotic microbiome [9]. Conversely, the achievement of a timely balance in the GIT ecosystem is a prerequisite for correct eubiosis in the subsequent productive phases of the pig.

Overall, any breach of even a single factor involved in the control of gut eubiosis could contribute to generate stress and discomfort that can concur to produce intestinal pain and impaired health of pigs. Natural evolution has provided swine with several servomechanisms for influencing the GIT community (innate and acquired immunity, adaptation of the mucosal interface, motility control, etc.). However, the evolution of the definition of the proper management rules of pig production has had and still has great relevance in influencing the context of the host to microbiome interaction. This evolution has recently found important tools in the availability of the so-called omics analytical technologies. The omics techniques, including next generation sequencing (NGS), have been applied to describe the contribution of some factors to the modulation of the gut microbial profile during early life of piglets. Several reviews already describe the evolution of the gut microbial profile in early life [8] and the main effect of diet [10,11,12,13]. However, further elucidation regarding the effect of several additional factors, including genetics, environmental and management stressors, live weight or physiological conditions, such as intrauterine growth restriction (IUGR), are still lacking. The authors hypothesize that a better description of the influence of these factors on the gut microbiota profile would benefit knowledge regarding the microbial and host interplay and the overall management of pig managing systems.

Thus, the aim of the present review is to highlight and describe the factors affecting the GIT microbial balance in young pigs during the pre- and post-weaning phases. This information represents a prerequisite to defining a road map for improving pig health and the production efficiency of the pig food chain.

## 2. Pre-Weaning

Piglets produced within an intensive production system are housed all together in pens and in contact with their mother’s feces, skin and mucosal surfaces until weaning; thus, it is expected that the microbiome of the newborn is shared with one of the other piglets in the litter and that it is largely dependent on the sow. Recent research in mice has evidenced the relevance of the vertical transmission of bacteria from the mother to the newborn. In fact, gut commensal bacterial lineages of wild mice from 17 inbred murine lines were vertically inherited for 10 generations in the laboratory. The opportunistic behavior of pathogenic bacteria to spread to different individuals was instead evidenced by the tendency towards their horizontal transmission [14]. In human newborns, the difference between gut microbiota in babies after natural delivery as compared to those after cesarean delivery supports the relevance of the natural mechanisms of vertical transmission. This has also been confirmed in pigs by the correlation between the sow vaginal microbiota and the piglet microbiota [15]. Overall, this demonstrates the practical importance of reducing the risk of deviating from this adaptation mechanism, which is presumably based on multiple factors and represented for the most part by the genetic background of the host and the microbial seeding acquired from the suckling environment.

Research has also demonstrated that the transgenerational effect on the gut microbiota could persist for a long time in the growing fattening phase with effects on the productive efficiency of the pig. Nevertheless, the complexity is a hallmark of the microbiota–host interaction. For instance, on the one hand, this is evidenced by changes in the gut microbiota composition after fecal microbiota transplantation from highly efficient finishing pigs to gestating sows and/or to their offspring in comparison with the control sows and their offspring [16]. On the other hand, this practice did not allow the transfer of favorable gut-associated traits, such as feed efficiency, which showed the importance of the characteristics of the receiving host as compared to the donor.

Considering the neonatal period to be an effective window of intervention is important for modulating the developmental dynamics of pigs, including the settlement of specific microbiota. In this section, the factors which play a role in modulating the GIT microbiota of suckling pigs are discussed.

### 2.1. Birth Body Weight and Physiological Maturation

The increase in the birth body weight of pigs is a key point which needs to be considered in order to globally improve the health of the litter. The increased number of pigs per litter due to the selection of highly prolific sows poses some major challenges in practice. Low birth body weight (LBW) pigs represent an intra-litter subpopulation which increases the complexity of properly prepare the pigs for weaning. As reported by Li et al. [17], the fecal bacterial community of LBW pigs (birth body weight (BW) 0.75−0.95 kg) presents a specific structure during the suckling period which is characterized by a lower relative abundance of Firmicutes at d3 and d7 after birth as compared with the normal birth weight (NBW) (birth BW 1.35∼1.55 kg). Interestingly, a lower level of Firmicutes has also been identified in the placenta of LBW infants [18]. Furthermore, LBW piglets are characterized by a lower relative abundance of *Lactobacillus*, *Streptococcus* and *Faecalibacterium* but a higher abundance of *Fusobacterium* at d3 and d7 after birth, and by a lower relative abundance of *Prevotella* at d21 after birth. In agreement with Li et al. [17], Gaukroger et al. [19] reported a lower relative abundance of *Lactobacillus*, unclassified *Prevotellaceae* and *Ruminococcaceae* UCG-005 in the feces of piglets presenting low average daily gains (ADGs) on Days 4, 8 and 14 after birth, respectively. More recently, differences have also been reported in the microbial composition of LBW piglets in the colon and ileum where the LBW piglets had a lower relative abundance of *Alistipes*, *Lachnospiraceae*, *Ruminococcaceae* and *Prevotellaceae*, respectively, in the different intestinal tracts [20]. These genera are well known for producing short chain fatty acids (SCFAs) which are characterized by exerting a beneficial effect on the host and for being a direct substrate for energy and for their immunomodulatory effects [21,22]. In fact, in the same study, lower levels of acetate and valerate on Days 7 and 21 and of propionate on Day 21 of age were observed in the colon of the LBW piglets.

A subcategory of LBW piglets is made up of intrauterine growth restriction (IUGR) piglets, defined as those in which the impaired growth and development of the embryo/fetus or its organs occurs during gestation [23]. According to the recent review of Jiang et al. [24], IUGR piglets could have a different microbial profile as compared with the NBW piglets, which could be related to maternal imprinting on the piglet gut microbial succession during gestation, at birth and during the suckling period. A study of Zhang et al. [25] has suggested that they had lower alpha diversity, lower relative abundance of Bacteroidetes and Bacteroides in the jejunum at Days 7, 21 and 28 after birth and lower *Oscillibacter* in the jejunum at d21 as compared with NBW pigs.

It is relevant to note that LBW pigs at birth have been associated with different microbial post-weaning profiles [17,20] and also as growing and adult animals [19,26]. For instance, the LBW pigs had a lower abundance of *Ruminococcaceae* UCG-005 but a higher abundance of *Ruminococcaceae* UCG-014 on Days 21 and 32, respectively [19] The IUGR pigs presented higher microbial diversity in the jejunum and ileum in the growing and finishing phases which has been associated with a lower performance [26]; at 25 kg of BW, they had a higher level of unclassified *Ruminococcaceae* in the ileum and lower *Ochrobactrum* abundance in the jejunum while, at 50 and 100 kg, they had higher Firmicutes abundance but lower Proteobacteria abundance in the jejunum and lower *Lactobacillus* abundance in the jejunum and ileum [26]. Overall, the differences in the microbial profile of LBW pigs could be associated with a different development of mucosal immunity since it is known that the intestinal microbiome and mucosal immunity are strictly related [27]. In fact, it is known that IUGR piglets have a higher intestinal permeability and a reduced intestinal barrier integrity [28,29], which could affect the establishment and succession of their intestinal microbiota as a higher number of bacteria adhering to the jejunal mucosa in LBW piglets as compared with the NBW piglets has been reported [30].

A lower birth weight can also affect growth in the first two weeks of life; this could be due to the feeding behavior and the competition for colostrum and milk for which the LBW piglets are disadvantaged as compared with NBW piglets, as observed by Morissette et al. [31]; overall, this could be a reason for a different microbial profile of 16-day-old pigs. Bacteria belonging to the Bacteroidetes phylum and the *Ruminococcaceae* family increased in piglets which grew faster while *Actinobacillus porcinus* and *Lactobacillus amylovorus* were reduced in pigs with slower growth.

### 2.2. Colostrum and Milk—Composition and Intake

Some phases of microbiota development can be seen in the guts of pigs from birth to 21–28 days of age, the time when weaning, in general, takes place. On the first day of life, the bacteria detected in the gut almost represent those immediately encountered in the environment of the piglet, being similar prevalently to the floor and the mother teat microbiota, and then milk or vagina (mainly Proteobacteria, *Clostridiaceae* and Firmicutes) [32,33,34,35,36]. More details on the environmental effect are given in Section 2.4. Then, in the suckling period, colostrum and mature milk provide a continuity base for the establishment of the gut microbiota. The colostrum phase is particularly associated with *Fusobacterium* and, even more so, to the *Bacteroides* genus [33,36]. Then, the *Lactobacillus* genus is more favored by the increase in lactose content with the passage from colostrum to mature milk [33], while *Ruminococcaceae* and *Enterococcaceae* families are also present [32]. Microbes degrading complex carbohydrates can also be found depending on the presence and the quality of solid feed supplements and the effective individual feed intake pattern. Of these, *Prevotella ruminicola* [37] is largely abundant and presumably explains part of the microbiota variability observed at this age. Changes also depend on the intestinal site [38]. Liu et al. [39] observed that harbored microbiota clustered with a milk microbial profile in the small intestine. Conversely, the microbiota of the large intestine was similar to that of the small intestine at birth, but it gradually diverged with age, resembling more and more the fecal microbiota of the sow. This indicated that both maternal milk and the fecal microbiome serve as microbial reservoirs for vertical transmission. The systemic and gut immune history of the mother is reflected in the composition and immunoglobulin profile of the colostrum, and it contributes to controlling the intestinal microbiota of the mother and their offspring [39]. However, no specific study is available regarding the effect of the quantity or the quality of immunoglobulins in colostrum or milk on the development of the gut microbiota of suckling pigs.

Moreover, the changes in glycosylation in the passage from colostrum to mature milk [40] modify the characteristics of the growth substrates of the gut microorganisms providing a partial explanation of the successive waves which contribute to shaping the complex gut microbiota community and its specific sugar digesting activity. It has been observed that the active substrates present in colostrum and milk, such as the galacto-oligosaccharides, can affect the intestinal microbiota of newborns, favoring the development of beneficial bacteria including *Lactobacillus* and unclassified *Lactobacillaceae* and reducing those belonging to *Clostridium sensu stricto* and *Escherichia* [41]. The presence of sialic acids and beta-exosamines stimulates the activity of the above strains, particularly those belonging to the *Bacteroides* genus which carry out sialidase and/or beta-exosaminidase activity [32]. The increased presence of fucosylated oligosaccharides observed with the maturation of sow milk gave more growth opportunity for the fucose-utilizing strains of *Enterobacteriaceae* inside the piglet gut microbiota [42]. Studies on human milk oligosaccharide composition and on its association with environmental factors have shown that the genetically determined ability to connect fucose to terminal galactose in an α1–2 linkage (defining the so-called a secretor or at the other extreme the non-secretor mother) could explain part of the specific sugar motif variability in milk [43]. Pigs also present a genetic variability for blood antigen groups which could be associated with the ability to add a specific link to the glycoproteins. Studies regarding pig colostrum have assessed the relevance of breed on the glycosylation of colostrum and its contribution to partially explaining the variability in maternal performance [44]. Furthermore, Meishan sows demonstrated a greater abundance of mannosylated-linked N-Acetylglucosamine oligosaccharide as compared to Large White sows, and, interestingly, another mannosylated-linked N-Acetylglucosamine oligosaccharide was positively associated with a strain of *Lactobacillus amylovorus* [45]. In general, this microbe is abundant in the swine intestine [46] and has probiotic properties [47].

From early studies, e.g. those by Elliott et al. [48] and Seerley et al. [49], it is known that the biochemical composition of colostrum and milk can be modulated by dietary intervention during pregnancy or lactation. However, the interest in assessing the effect of sow feeding on the gut microbiota of offspring by means of the changes in the milk or the colostrum profile is only recent. The administration of yeast-derived products (YDPs) to sows during gestation and lactation increased the fat percentage of colostrum as well as increased the abundance of *Roseburia*, *Paraprevotella*, *Falsiporphyromonas*, *Eubacterium* and *Alkalitalea* in their feces. On the contrary, the prevalence of *Turicibacter*, *Papillibacter*, *Helicobacter*, *Escherichia/Shigella* and *Desulfovibri* decreased. The one-week-old pigs raised with the sows fed with YDPs had feces with a higher abundance of Firmicutes and a low abundance of Bacteroidetes as well as having a greater proportion of the genera *Oscillibacter*, *Clostridium* IV, *Blautia*, *Gemmiger*, *Anaerobacterium*, *Anaerovibrio* and *Paraprevotella* as compared with the controls. The differences persisted in four-week-old pigs, which showed decreased abundances of Spriochaetes and Synergistetes and higher abundances of Actinobacteria and Lentisphaerae [39]. In addition to the feed additive, nutrients play a role in modifying the sow metabolism as well as its microbiota. The administration of the milk fat globule membrane during the last month of gestation improved fecal *Prevotella* in the sows and increased microbial diversity and richness in the piglets [50]. On the other hand, feeding pregnant sows with resistant starch altered biochemical colostrum composition as well as maternal microbiota, but did not affect the piglets’ microbial profile [51]. Conversely, feeding gestating-lactating sows with a diet based on wheat bran not only increased the lactose content of the milk, but also affected the colon microbiota of the offspring [52]. This latter effect was not dependent on the fecal microbiota composition of the mothers because it was affected differently by the diet [52]. Thus, it cannot be excluded that the variation in the lactose content of the milk, which was induced by the diet, was directly effective in changing the piglet intestinal microbiota. Furthermore, it is possible that the different composition of colostrum and milk selects microbe variants with quite a different affinity for nutrient, without changing the main microbiota composition. Unfortunately, no data of sequencing of the whole genome or on microbial phenotyping of piglets fed suckled by sows with different colostrum or milk composition are available.

### 2.3. Genetics Background

It is known, mainly from studies on humans and mice, that, of the different factors, the host genetics contribute to affecting the establishment of the intestinal microbiota. In pigs, although studies are still at an initial stage, some attempts have highlighted differences in the intestinal and fecal microbial profile among different pig breeds in adult pigs [53,54]. Chinese Jinhua pigs are characterized by a greater abundance of Firmicutes (70.4%) as compared with western breeds, including the Duroc, Yorkshire and Landrace breeds, which are characterized by a lower level of Firmicutes (39.6–45.6%) and a higher level of Bacteroidetes (47.6–57.0%) [53,55]. Furthermore, few studies have shown the heritability of specific taxa, which vary from low to high values [34,56]. However, little is still known regarding newborn and pre-weaning piglets.

Since the maternal environment tends to be a key factor in determining early-life gut microbiota colonization, some studies, using cross-fostering experiments, have investigated whether the piglet microbiota is mainly influenced by the mother-genetics or by the mother-environment. The studies found that different breeds of piglets showed certain identical microbial taxa in the cecum content [57] and feces [45,58] when fostered by a nursing mother of a given breed, also resulting in a difference in the expression of the interleukin 10 gene in the colon [45]. These studies pointed out that, even if the effect of the mother-environment was more consistent, genetics played a significant role in shaping the fecal microbial profile of piglets and that it could be associated with host robustness.

The application of selection indices which directly or indirectly include the survival of piglets, such as the body weight of piglets at d21, may contribute to better resilience of the gut microbiota, thanks to the genetic selection of piglets with improved health conditions [59]. However, the performance and health status of pigs are affected by multiple environmental factors, and, as discussed by Guy et al. [60], several variables need to be considered simultaneously, including genotype, disease and descriptors of the environment and their interaction in the design of selection indices. The growing knowledge of the pig genome and the inclusion of some specific markers related to the interaction between the host and the intestinal microbiota in the pig selection markers into breeding schemes should be considered in order to increase the explanation of the performance and health status of piglets and the reduction of the risk of gut dysbiosis.

### 2.4. Environmental Stressors and Management

It is known that the environment plays a pivotal role in the microbial ecosystem settlement even at an early age and influences it for a long period of time. The neonatal birth environment provided 2–10% of the mucosal microbiota in the large intestine within the first two weeks of piglet life, and its contribution is then quickly reduced by age [61]. The microbial profiles of the vagina, colostrum and feces from sows were similar to each other and were highly correlated with the piglet bacterial profile. In fact, the bacterial genera present in the GIT of the piglet had a strong correlation with the bacterial genera present in the vaginal and fecal samples and the colostrum of the sow [62]. Liu et al. [61] reported that vaginal microbiota contributed to 6–16% of mucosa-associated microbiota in the ileum, cecum and colon in a one-day-old pigs. However, this is a transient effect which is strongly diminished by Day 35.

For instance, the effect of cohabitation from Day 3 of artificially-fed piglets is observable in one- or two-week-old piglets but it is the strongest at either three or four weeks post-birth, while the litter of origin was not important [63]. More recently, it has been observed that environmental microbiomes, including those from slatted floors and nipple surfaces, can significantly influence the fecal microbial profile of newborn piglets, without however having a long-term effect [33]. Conversely, it seems that the microbiome from the mother can stabilize the colonization of the intestine of a newborn with a co-occurrence effect, but with more involvement of the suckling milk environment [33,34,35] and less the mother’s feces [36] or vagina [35].

Nevertheless, even if the effect of the environmental microbiome does not seem to be persistent over time, it could be particularly relevant in newborn piglets, especially in an intensive rearing system in which they are exposed to several risks and stressful factors, including hypothermia, high teat competition, mutilation and mixing of the litter by means of the split suckling and cross-fostering techniques [64]. It is known that the gut microbiome is strictly connected to the behavior of the host since it communicates with the brain by means of the production of a variety of hormones and neurotransmitters in the so-called “microbiota–gut–brain axis”. Thus, these stressful conditions can profoundly affect the gut microbial composition, as previously reported by Foster et al. [65]. Of the stressor conditions, cross-fostering is commonly applied in pig rearing. However, its effect on the microbiota composition of the piglet in the subsequent suckling period appears to be scarce. In a cross-fostering model [62], no specific cluster of piglets undergoing cross-fostering treatments was observed regarding the GIT microbiome. Only the abundances of *Treponema*, *Campylobacter* and *Tannerella* genera were higher in the feces of 21-day-old piglets exchanged at birth to receive colostrum and post-colostral milk from a foster sow and which remained with the foster sow as compared with piglets constantly suckled by their own mothers [62], while the *Escherichia/Shigella* genera were higher in pigs receiving colostrum from fostering sows and post-colostral milk from their own mother [62].

Considering the sow–piglet vertical transmission of the microbiota, distressing the sows by altering their microbiota can directly affect that of the litter. In fact, heat stress (HS) in late gestation affected the beta diversity of the intestinal and vaginal microbiota of the sows and, thus, that of the colon of the offspring [66]. The microbial communities in HT sows shifted toward a type less oriented to the digestion capacity of plant oligosaccharides [66]. These sows transmitted to the offspring more bacteria of *Escherichia-Shigella* genus from the mixed intestine vagina path and of *Fusobacterium* from the sole vagina. Furthermore, the same authors observed that maternal HS increased serum adrenocorticotropic hormone (ACTH) in the offspring, indicating the activation of the hypothalamus–pituitary–adrenal axis, confirming that the transmission of stress signals during fetal life affected the early life physiology of piglets. However, little is still known regarding the connection between routinely stressful factors and the microbiome of the piglets, and more studies are needed to investigate this.

It is widely recognized that the adoption of certain management practices can reduce specific stress during suckling. The administration of milk replacer is a common practice to reduce the frequency of underfed pigs due to the competition for the teat, especially in those farrowed from highly prolific sows. In suckling pigs fed sow milk, the taxonomy of the gastrointestinal microbiota clearly clustered as compared to that obtained from piglets fed bovine milk or bovine colostrum [67]. The stomachs of sow-milk-fed piglets had a higher abundance of Lactobacillus, while those fed the milk replacer and bovine colostrum were characterized by *Enterobacteriaceae* and *Lactococcus*, respectively. *Enterobacteriaceae* were also more abundant in the ileal digesta of the piglets receiving the milk replacer. Sow-milk-fed pigs had a higher abundance of the *Blautia* genus in the mid-colon [67].

Improvements in farrowing and lactation have the possibility of ameliorating welfare outcomes for both piglets and sows and could result in positively affecting the intestinal microbiome of the offspring. The careful use of sanitization measures is a founding pillar for the above. For instance, the transmission of environmental pathogens could be limited by the washing of the sows before their entrance into the farrowing rooms with no negative effect on the transmission of the gut-based microbiota to the newborn, if it is carefully done with mild solutions. However, despite the fact that the washing of the sows is included as a measure to improve the biosecurity around farrowing [68], the authors were not able to find any study putting this practice in association with the variation of the gut microbiota profile of the offspring.

On the other hand, excessive hygienic conditions and limited microbial exposure during early life have shown significant impairment in the microbiota of adult pigs. In particular, even if excessive hygienic conditions can ensure a higher diverse microbiota in early life, they can reduce the level of Firmicutes and increase those of Bacteroides and Proteobacteria, hindering the progression towards an adult-type gut microbiota [69]. Trials in antibiotic-treated mice have indicated that upsetting the gut microbiota favors pathogen multiplication, such as for Salmonella infection, later in life [70].

### 2.5. Antimicrobials

During suckling, the use of antibiotics should be limited in order to ensure the establishment of the intestinal microbiota and since piglets should be protected by the passive immunity provided by colostrum and milk. However, there are some specific conditions which increase the risks of health impairment during suckling; of these, the prevalence of pigs with an initial LBW should be considered since, as described previously, LBW piglets are characterized by a different intestinal microbiome, a higher incidence of mortality and a lower long-term growth potential. In addition, some procedures, such as castration and tail docking, can predispose infection with a consequent need for antibiotic administration.

Antibiotic administration to piglets in early life has been shown to have a long-term effect, for at least five weeks, on both the diversity of the microbiota, which is reduced, and on its composition [71,72,73]. For instance, the intramuscular injection of 2.5 mg/kg body weight of Tulathromycin at Day 4 of age increased the abundance of all anaerobic bacteria, including *Bifidobacterium*, *Eubacterium*, *Faecalibacterium prausnitzii* and *Solobacterium moorei*, and reduced the abundance of facultative anaerobic bacteria, such as *Streptococcus aureus* [72]. Janczyk et al. [71] observed a decrease in both diversity and richness in the ileal microbiota of 39-day-old piglets which had been administered a single dose of amoxicillin intramuscularly at birth. From an ecological perspective, a reduction in alpha diversity indices has been associated with a less mature and stable microbial profile since a decrease in bacterial species in the community would reduce the functional redundancy, which would be crucial in contrasting stressful events, such as weaning [74].

Furthermore, it has been observed that a change in the microbial composition by means of early-life antibiotic treatment also significantly modified the related immune processes and the digestion and absorption of the nutrients [72,75,76].

Finally, a study on humans has suggested that maternal antibiotic treatment can also influence the early gut microbiome of their offspring by altering the vaginal microbiome [77]. In fact, amoxicillin administered in late gestation affected sow vaginal [78] and fecal [78,79] microbial diversity. This could have inconsistent results on the piglet microbiome, since Arnal et al. [79] observed a significant effect on the microbiota of the small intestine of the offspring but not of the colon and de Greef et al. [78] did not observe any effect on the jejunal digesta microbiome. Some effects of maternal antibiotic treatment on the gut physiology and morphology of the offspring have been seen in early-life [78,79,80,81], but, for some response parameters, also later in life (169 days of age) [78,79,80]. Thus, this aspect is worth being investigated more in-depth in pig, and a potential effect on the newborn microbiome and following eubiosis of the growing pig cannot be excluded.

### 2.6. Diet

The first aim of complementing milk with a creep feed was mainly to anticipate the ability of piglets to secrete the digestive enzymes needed to digest solid feeds and to get them used to eating them [82,83]. Nevertheless, the new feed matrix could also stimulate the seeding of microorganisms capable of using it. In general, the suckling of milk has a stabilizing effect on gut health for its constituents, and often no effect of providing creep feed is seen on the main GIT microbial markers (Lactic acid bacteria, coliforms) as well as on the SCFAs [84]. In addition, the consumption of creep feed before weaning did not reduce the prevalence, duration or severity of the diarrhea induced experimentally by Enterotoxigenic *Escherichia coli* (ETEC) F4 before weaning or after weaning [85]. Unfortunately, since the development of modern methods based on microbial sequencing, research regarding the effect of providing creep feed provision to suckling pigs on the gut microbiome has been scarce. However, it has been shown that the different conditions of piglets at the time of the provision of the creep feed can affect the response of the GIT microbiota. A relevant negative correlation between *Prevotella* and *Escherichia* was observed in healthy piglets supplemented with creep feed from Week 3, while, in diarrheic piglets receiving the same treatment, this correlation was relatively weaker [86]. It can also be hypothesized that the quality and the palatability of the creep feed affects the development of the GIT microbiota as a direct substrate. The provision of a milk-rich creep feed, a starter mixture or a corn–soybean-based mixture to suckling pigs from Day 14 of age did not affect the presence of bacterial RNA from selected microorganisms (*Escherichia coli K88/Lactobacillus casei/Lactobacillus plantarum/Bacillus subtilis*) in the ileal, cecal and colic content obtained on Day 4 post-weaning [87], as was also the case for the number of pigs consuming creep feed, the creep feed intake of the litter and the live weight on Day 35 post-weaning. The effect of increasing the fiber content of standard creep feed with approximately 0.8% neutral detergent fiber from alfalfa hay, wheat bran or cellulose was studied in suckling pigs from 7 to 22 days of age [37]. At this final age, the microbiota profile of the large intestine changed according to the different fiber additions, and, particularly with alfalfa, the presence of *Streptococcus suis* was reduced [37]. Again, it can be stated that more research is needed to understand the relevance of the quality and timing of creep feed supplementation in order to achieve an optimal condition of the GIT microbiota in the subsequent lifetime of the swine. In addition, the availability of data of sequencing of the whole genome of piglets fed with or without creep feed would provide more understanding on the activation of different microbial digesting ability, not only associated with their 16S rRNA gene variations.

## 3. Weaning and Post-Weaning

In intensive pig production, the post-weaning period is a critical stage in the animal’s life. Even if strategies are adopted during the suckling period to improve the host maturation, pigs have not completely developed enough to digest vegetable substrates or to achieve immune competence at weaning (28 days of age). This condition, together with the so-called “weaning stress” due to a plethora of social and environmental changes, can predispose the piglets to dysbiosis [88]. As reviewed by Gresse et al. [88], weaning transition is characterized by a loss of microbial diversity, a decrease in the abundance of bacteria belonging to the Lactobacillus group and an increase in the abundance of facultative anaerobes, including bacteria belonging to the *Enterobacteriaceae*, *Proteobacteriaceae*, *Clostridiaceae* and *Prevotellaceae* families [74,88].

The aim of this section is to identify the most important risk factors which could predispose an imbalance of the gut microbiota at weaning and to describe their effect on the gut microbial profile.

### 3.1. Age and Live Weight at Weaning

When the modern domestic pig is reared in a wild environment, weaning is a naturally slow progressive event following the reduction of milk available from the mother and the increasing amount of solid feed the young pigs can forage [89,90], this process ending between 12 and 17 weeks of age. Physical distancing from the mother also progressively increases [90]. This is assumed to assure the evolutionary defined stabilization of the future adult GIT eubiosis. Under free range conditions, the huge variability of microorganisms in the environment, including soil bacteria, is expected to couple with high variable seeding in the gut microbiota. However, experimentally, the gut microbiota variability in pigs reared until weaning in an outdoor system was reduced to up to 56 days of age as compared to pigs of the same genetic line reared under isolated conditions [91]. This did not preclude the flourishing of a beneficial GIT bacterial pattern (Lactobacilli in particular), while it favored better control of pathogenic species [91]. However, to the best of the authors’ knowledge, studies extending the observations regarding the effect of natural weaning on the GIT eubiosis later in pig life are not available.

In intensive pig production, a weaning schedule is economically sound when it is capable of producing healthy piglets with a good feed intake and physiologically mature enough to digest a solid diet so as to develop lean meat. The majority of the scientific studies carried out in the era of the diffuse use of antibiotics in livestock have agreed with the practice of weaning pigs at approximately 21 and 28 days of age [92]. This indication was based on knowledge targeted to maximize the reproductive performances of sows, the lactation length being optimized. However, within this age range, it has already been observed that delaying the weaning age to 28 days assures better control of the transient inflammation related to the weaning phase [93] and a reduction in mortality within seven weeks post-weaning [94]. Curiously, with the increasing need to limit the use of antibiotics, there has not been a rethinking of the preference for early weaning in order to optimize the preparation of the mature GIT microbiota, at least as far as published research is concerned. Considering the relevance of the specific classes of weight of the piglets at a given age was a first step in this direction. Delaying the weaning age by one week to an average of 32 days benefitted the performance of lighter pigs when reared to a common final weight (20 and 60 kg) [95]. Pigs weaned later, whatever the weight class, also had reduced mortality. This may also have been related to a different GIT microbiota pattern, but this was not considered in that study.

However, there is the need for defining the target profiles of the ideal mature GIT microbiota in order to optimize weaning age. Follow-up studies regarding pigs under both genetic selection and standard rearing have indicated that the presence of bacteria capable of degrading complex carbohydrates, including fiber degrading organisms, is the hallmark of a mature microbiota in the colon or feces (growing-finishing pigs), including the *Prevotella* and *Treponema* genera [95,96,97,98,99]. Furthermore, the operational taxonomic units (OTUs) assigned to the *Prevotella* genus were genetically associated with growth performance during different growing phases [100]. It should also be considered that pigs in the growing-finishing phases are, in general, reared in more stable groups and with dietary formulations given for longer time periods. This also favors animals which have a less variable GIT microbiota, as indicated by the reduced microbial alpha diversity in individual pigs having a better growth performance in these phases [98], presumably thanks to a reduced incidence of costs related to the immune system activation. Overall, this indicates the complexity of predicting the long-term response based only on observations carried out immediately after weaning [98].

### 3.2. Management

The current weaning practice breaches the contiguity of the gut community owing to the removal of contact with the mother and her excreta, the transient anorexia of the newly weaned pig and the massive presence of diverse chemical structures entering the gut. The recovery of the pre-weaning microbial community may require time; however, research, in general, indicates that the link between the pre- and post-weaning microbiome is limited [38]. Nevertheless, it can be hypothesized that the gap can be minimized by opportune rearing methods. No direct comparison was found in the literature regarding this; however, in one study [63], one- or two-week-old siblings showed a more homogeneous gut microbiota than other contemporary piglets, while, after 31 days of age, the gut microbiota was associated between cohabitants, but not between siblings. This indicated the ability of the gut microbiota inside the group to converge toward a common profile. Furthermore, the practice of keeping weaned pigs by litter will apparently contrast later in the growing-fattening phase with the need to feed males and females according to their different body growth; however, the new techniques of precision feeding could contribute to resolving this problem. Moreover, this also implies that pigs of very different weights should be kept together, and, in general, this could create some time-consuming problems for management by the farmer. It is possible that future precision feeding tools could resolve this problem. On the whole, more studies to test the long-term advantages or drawbacks of maintaining littermates together for the following growing-fattening periods are urgently needed. The observation that piglets stressed by mixing were more likely prone to Salmonella infection validates the need for confirming the advantages of a more prudent post-weaning grouping method [101].

### 3.3. Genetics Background

Since pigs are subject to a number of stressful factors which contribute to generating an imbalance in the intestinal microbiome during weaning transition and which can result in post-weaning diarrhea, a potential strategy could be to select pigs for their robustness and to include markers related to the host-microbiota cross-talk in the genetic indices. Two main genetic strategies could be implemented; one more specific strategy would be to select pigs genetically resistant to the main pathogens which may be at high risk of developing post-weaning diarrhea, and a second more general strategy would be to select pigs genetically more resilient by having a more responsive immune system.

Concerning the first proposed strategy, some genetic markers associated with resistance to ETEC F4 and F18, considered two of the main pathogens responsible for the post-weaning diarrhea, were identified and summarized by Luise et al. [102]. These host genetic markers have been additionally associated not only with the resistance of specific pathogens but also with the fecal and intestinal microbial profile of post-weaning piglets [102,103,104,105]. For instance, a Single Nucleotide Polymorphism (SNP) mutation located on the Fucosyltransferase 1 (*FUT1*) gene which has been associated with susceptibility to ETEC F18 significantly influenced both the alpha and the beta diversity indices in healthy piglets [104]. The genotype associated with ETEC F18 resistance was discriminated by OTUs belonging to the *Lactobacillus* genus while the susceptible genotype was characterized by a higher abundance of *Veillonella*, hemolytic bacteria and Enterobacteriaceae [104,105]. As suggested by the authors, the contribution of *FUT1* influencing the gut microbial profile could be explained by the role of the gene as it is involved in the glycosylation on the protein structure of the porcine small intestinal mucosa, as observed by Hesselager et al. [106].

Concerning the second proposed strategy, to select pigs having a better responsive immune system, some genetic variants linked to the widest cross-talk between host and bacteria could be of interest. In this context, genes related to the pattern recognition receptors (PRRs) of the small intestine including Toll-Like Receptors (TLRs) and NOD-like receptors (NLRs) could be considered since they are significantly involved in the host–microbiome cross-talk, especially during weaning transition [107]. Recently, the study by Xiao et al. [108], which applied fecal transplantation (FT) in post-weaning piglets of two divergent breeds, such as Yorkshire (high growth rate but more susceptible to disease) and Tibetan (less selected for growth rate and more robust), showed that FT could significantly affect the microbial profile and the regulation of innate immune responses; however, the breed effect was significantly higher and the more robust breeds harbor a specific gut microbiome which activates the PRRs.

A first attempt at estimation of the genetic parameters of the gut microbiota composition linked to immunity traits was carried out by Estellé et al. [109], who identified both a positive and a negative correlation between *Dialister*, *Prevotella* and *Roseburia* and the blood values of monocytes, eosinophils, platelets, hemoglobin and red blood cell parameters in a selected population of 533 post-weaning piglets obtained from 90 families. Furthermore, it is interesting to note that both *Dialister* (h^2^ = 0.22) and *Prevotella* (h^2^ = 0.45) showed medium-high heritability.

Overall, it is clear that host-genetics can play a role in piglet response to the gut microbiota, including both commensal and pathogenic bacteria, and, in turn, it can modulate the gut eubiosis of post-weaning pigs. The growing knowledge of the pig genome and pig intestinal microbiome, and the potential inclusion of some specific markers related to the interaction between host and bacteria in pig selection (e.g., resistance to *E. coli* infection), could contribute to reducing the risk of the gut dysbiosis typical of the post-weaning phase or, more in general, to increasing piglet robustness.

### 3.4. Post-Weaning Diet

Diet is one of the main factors capable of modulating the gut microbial profile; thus, several reviews dealing with the effects of post-weaning diets on microbiota have been published [10,11,12,13]. Feeding measures of the post-weaning phase should contribute to reducing the gap between the pre- and the post-weaning phases, and, focusing on the gut microbiota, these should promote and restore the intestinal microbial eubiosis which was lost with weaning [88]. In this latter context, the main objectives that a post-weaning diet should satisfy are summarized in the following points: (1) restore the previous commensal bacteria, such as *Lactobacillus*, and restore the bacterial richness which was lost with weaning; (2) favor the development of bacteria capable of using the nutrients of a growing diet, such as structured carbohydrates; (3) favor the development of bacteria which can have a beneficial interplay with the host and promote the development of a host mucosal immune system; and (4) render disadvantageous the proliferation of non-beneficial and pathogenic bacteria which significantly increase immediately after weaning.

An efficient way of modulating the intestinal microbiota is to regulate the nutritional composition of the diet, including the content of protein, starch and fiber [12,110,111]. As for other mammals, it has been well established that the ratio and type of protein and fiber play a key role in the modulation of the gut microbiota, affecting the development and establishment of beneficial (lactic acid bacteria) or opportunistic/pathogenic bacteria, which, in turn, can affect the intestinal health of the host [112,113]. An excess of dietary protein concentration can promote the proliferation of pathogenic bacteria which prefer protein as an energy source, thus increasing the protein intestinal fermentation in the large intestine. It has been hypothesized that a reduction in dietary protein and the use of more digestible proteins can therefore affect the microbial composition. Studies have proven that a reduction of the crude protein content in the feed formula resulted in an increase of the microbial diversity in both the small and the large intestine; the promotion of *Lachnospiraceae*, *Prevotellaceae* and *Veillonellaceae* in the large intestine; and reduction of the abundance of non-beneficial bacteria, including *Streptococcaceae* and *Enterobacteriaceae*, in the small intestine [114,115]. Dietary fermentable carbohydrates represent a suitable substrate for the microbiota; thus, they can play a crucial role in modulating the intestinal microbiota of post-weaning piglets [116,117]. In this context, as reviewed by Willams et al. [117], both soluble and insoluble fiber can modulate the microbial population; e.g., according to Molist et al. [118] and Gerritsen et al. [119], the increase in insoluble fiber can reduce the population of potentially pathogenic bacteria, including *E. coli*. More recently, Chen et al. [120], comparing the effect of soluble and insoluble fiber on the colonic bacteria of post-weaning piglets, suggested a more extensive effect of soluble fiber.

Apart from nutrients, a frequent strategy for restoring and supporting microbial eubiosis in the post-weaning phase is to supplement the diets with additives having the target of attenuating the gap between the pre- and post-weaning microbiota and enhancing the colonization of beneficial bacteria in the GIT ecosystem. In this context, probiotics have been widely studied and the main results have been summarized in targeted reviews [121,122,123]. Since the topic is extensive, in this review, the most recent studies regarding the probiotics which have reported a positive and promising effect on the modulation of the microbiota have been summarized (Table 1). Feeding the *Lactobacillus amylovorus* strain, which is dominant in the pre-weaning pig microbial community, could help in maintaining its presence in post-feeding and contributing to protecting the post-weaning piglets from *E. coli* F4 infection [46]. In agreement with Konstantinov et al. [46], another lactic acid bacteria (LAB), *Lactobacillus plantarum*, has shown promising results, contributing to increasing the level of recognized beneficial bacteria, including bacteria belonging to the *Bifidobacteriaceae* family [124] and the genera *Lactobacillus* and *Mitsuokella* [125,126]. More recently, attention to other bacteria, in addition to LAB bacteria, has increased and, of them, *Bacillus* spp. probiotics have been proposed as suitable feed additives owing to their ability to tolerate harsh environmental conditions and to germinate in the guts of animals [127]. In post-weaning piglets, the *B. subtilis* species has especially shown promising results in the modulation of the gut microbiota [127,128,129], reducing non-beneficial bacteria, such as *Enterobacteriaceae* [130], promoting beneficial bacteria (including *Coprococcus* and *Bifidobacterium*) which can improve hindgut health by influencing the SCFAs and the branched-chain fatty acids (BCFAs) profile [128] (Table 1). Furthermore, *B. subtilis* promoted the ileal villi height, the turnover of the epithelial cells and the gene sets related to intestinal absorption and secretion activities resulting in reduced diarrhea due to *E. coli* F4 and F18 challenges [128,130]. An additional promising probiotic for livestock is the yeast *Saccharomyces cerevisiae* (*S. cerevisiae*). *S. cerevisiae* has been considered to be not endogenous in the gut microbiomes of humans and animals, but this has recently been under more in-depth investigation [131]; in addition to the fact that an intermittent supply of *S. cerevisiae* from birth to weaning significantly affects the structure of the post-weaning piglet gut microbiota and promotes the development of specific bacteria, including those belonging to the *Ruminococcaceae*, *Clostridiaceae*, *Peptostreptococcaceae* and *Peptococcaceae* families, it contributes to an increase in piglet growth performance [132].

An additional feeding strategy which has recently aroused interest due to its potential effect in promoting the gut health of post-weaning piglets, via microbial modulation, is represented by organic acids. Organic acids have been widely used for decades in pig feed as a preserver of feed nutritional qualities [133]. Previous reviews have analyzed the effect of several single and mixed organic acids on post-weaning microbial composition [134,135]. The effects of organic acids in the modulation of intestinal microbiota in post-weaning piglets have been mainly ascribed to their capacity to reduce the pH of the stomach. The gastric environment plays a pivotal role in the modulation of intestinal environments since it acts as an ecological filter, reducing the proliferation of bacteria sensitive to a low pH and acid resistant microbes, including lactic acid bacteria [135,136]. The most recent results of the most common organic acid supply on the gut microbiota of post-weaning piglets are reported in Table 1. The effect of organic acids on specific taxa abundancy varies depending on the organic acid type and dose and the intestinal site analyzed. For instance, 1.4 g/kg of formic acid reduced the abundance of *Streptococcus* in the jejunum which was, instead, increased by 6.4 g/kg of formic acid. Contrary to the study of Luise et al. [137], Ren et al. [138] found no effect on the ileal microbiota of post-weaning piglets fed with a diet which supplied 0.60% of formic acid. A significant and consistent effect on the taxa modulation in both the ileum and the colon was observed by Huang et al. [139], in a study in which a diet supplemented with 1 g/kg of encapsulated sodium butyrate coupled with 50 mg kitasamycin/kg and 20 mg colistin sulfate/kg significantly improved the abundance of *Clostridiaceae* and reduced that of *Lactobacillaceae*.

In addition, it could be suggested that organic acids could modulate the intestinal microbial profile and structure (alpha and beta diversity), as observed by [137,139,140]. Some attempts to evaluate the effect of acidifiers in not only modifying the taxonomy but also the functionality of the microbiota were made by Zhai et al. [140] and Ren et al. [138] using the prediction of metabolic pathways with PICRUSt and Tax4Fun. Zhai et al. [140] observed that protected benzoic acid enhanced the degradation of caprolactam, a secondary metabolite of benzoic acid, C5 branched dibasic acid metabolism and amino acid metabolism (tryptophan) in the cecal microbiota. Pathways related to amino acid metabolism were also confirmed to be affected by Ren et al. [138] in the ileum of piglets supplied with formic acid. However, as already reported by Zhai et al. [140], there is the need of whole-genome shotgun sequencing and more accurate analysis to fully clarify the effect of acidifier on the functionality of gut microbiome. Thus, additional investigation is needed to better associate the beneficial effects which the organic acids supply has on the growth and health of post-weaning pigs with gut microbial modulation.

It is known that diet is one of the main factors affecting the microbial profile, there are several reviews dealing with feeding strategies, especially regarding feed additives as gut microbial modulators [117,134,135,141]. However, to avoid redundancy with other reviews specifically focused on nutrients, feed additives and feeding strategies as gut microbial modulators, the authors present an overview of the most promising feeding interventions which modulate the gut microbiota. Therefore, the authors highlight the above-mentioned studies to demonstrate the effectiveness of targeted feeding strategies in limiting the loss of microbial eubiosis which generally occurs at weaning.

**Table 1:** Summary of the effects of probiotics and organic acid on the pig microbiota of post-weaning piglets.

Intervention	Quantity	Phase	Analyzed Matrix	Alpha Indices	Beta-Diversity	Difference in Taxa	Direction	Reference
Probiotics								
*Bacillus subtilis* *KN-42*	2×10^9^; 4×10^9^; 20×10^9^ CFU/g	Post-weaning period. From weaning for 28 days	Feces	↑ microbial diversity in 4 × 10^9^ diet	Not tested	*Lactobacillus*	↑ in 4 × 10^9^ diet	[142]
						*E. coli*	↓ in 4 × 10^9^ diet	
*Bacillus subtilis DSM25841*	1.28 × 10^6^ CFU/g	Post-weaning period. From weaning for 21 days	Cecum	=	=	*Enterobacteriaceae*	↓	[130]
*Bacillus subtilis* *DSM 32315*	5 g/kg of 2 × 10^9^ CFU/g.	Post-weaning period. From weaning for 21 days	Jejunum	=	=	*Leucobacter* and *Cupriavidus*	↑	[128]
			Ileum	=	≠	*Thermus*, *Coprococcus* and *Bifidobacterium*	↑	
			Colon	=	≠	*Succiniclasticum*	↑	
*Bacillus subtilis* *DSM 32540*	0.5 g/kg (1 × 10^9^ CFU/kg)	Post-weaning period. From weaning for 28 days	Jejunum	=	≠	=	-	[129]
			Ileum	=	≠	*Lachnospiraceae*, *Peptostreptococcaceae* and *Pasteurellaceae*	↓	
			Colon	=	≠	=	=	
*Saccharomyces cerevisiae*	3 g/kg of 4.5 × 10^6^ CFU/g	Post-weaning period. From weaning for 41 days	Feces	=	Not tested	=	=	[143]
*Saccharomyces cerevisiae* (Actisaf Sc 47)	2.5 × 10^10^ CFU/piglet	From birth to 28 days post-weaning	Cecum	=	≠	*Coriobacteriaceae*, *Halanaerobiaceae*, *Peptococcaceae*, *Peptostreptococcaceae*, *Ruminococcaceae* and *Turicibacteraceae*	↑	[132]
			Colon	=	≠	*Coriobacteriaceae*, *Halanaerobiaceae*, *Peptococcaceae*, *Peptostreptococcaceae*, *Ruminococcaceae* and *Turicibacteraceae*	↑	
*Lactobacillus plantarum PFM105*	2 × 10^7^ CFU/g	Post-weaning period. From weaning for 21 days	Colon	↑ Simpson	=	*Bifidobacteriaceae*	↑	[124]
*Lactobacillus plantarum*	3·5 × 10^10^ CFU/g	Post-weaning period. From weaning for 14 days	Feces			*Clostridium*_*sensu_stricto*_1, *Parabacteroides* and *Ruminococcus*_1	↓	[126]
						*Lactobacillus*	↑	
*Lactobacilus plantarum* (JN560899.1)	10^9^ CFU/kg	Post-weaning period. From weaning for 28 days	Feces	=	Not tested	*Selenomonas* and *Mitsuokella*	↑	[125]
Organic acids								
Formic acid	1.4 g/kg or 6.4 g/kg	Post-weaning period. From weaning for 42 days	Jejunum	↑ Chao1 (6.4 g/kg)	=	*Gemella, Lactobacillus, Parvimonas*	↓ (1.4 g/kg) ↓ (6.4 g/kg)	[137]
						*Streptococcus*	↓ (1.4 g/kg) ↑ (6.4 g/kg)	
						*Turicibacter*	↓ (1.4 g/kg)	
						*Acinetobacter, Fusobacterium, Leuconostoc*	↓ (6.4 g/kg)	
Formic acid	0.60 g/kg	Post-weaning period. From weaning for 8 days	Ileum	=	=	=	=	[138]
Benzoic acid	2 g/kg + essential oils	Post-weaning period. From weaning for 28 days	Colon	=	≠	Bacteroides, Unclassified S24-7	↑	[140]
						*Prevotella*	↓	
Sodium butyrate (encapsulatded)	1g/kg + antibiotic	Post-weaning period. From weaning for 28 days	Colon	↑ Shannon	Not tested	Bacteroidetes, *Clostridiaceae*, *Ruminococcaceae*, *Lachnospiraceae*	↑	[139]
				↓ Simpson		*Lactobacillaceae*	↓	
			Ileum	=	Not tested	*Clostridiaceae*	↑	
						*Pasteurellaceae*, *Lactobacillaceae*, *Enterobacteriaceae*	↓	

### 3.5. Antimicrobials

In general, antimicrobials are used on livestock for a number of reasons: (1) as therapeutics; (2) more commonly as metaphylactics, meaning that the presence of clinical illness in one animal triggers drug treatment of the whole herd or flock; (3) as prophylactics; and (4) for growth promotion. A ban on the use of growth promoters was implemented throughout the EU in 2006 and a voluntary ban has recently been introduced in the USA. However, this has prompted compensatory increases in metaphylactic and prophylactic use.

In pigs, the majority of antibiotics are used in the immediate post-weaning phase to prevent bacterial diseases facilitated by the weaning transition. As observed for the suckling period, in the post-weaning period, antibiotics can affect the gut microbial profile in the mid- and long term. For instance, Massacci et al. [144] observed that parenteral (15 mg/kg bodyweight two administrations at a 48-h interval) and oral (12–20 mg/kg bodyweight of the suspension orally twice a day for five days) administration of amoxicillin reduced the fecal microbial diversity of weaning piglets in agreement with Thymann et al. [145], who found that intramuscularly administered amoxicillin coupled with in feed ZnO administration reduced the microbial diversity in the small intestine. Furthermore, according to Massacci et al. [144] and Connelly et al. [146], the oral administration of amoxicillin strongly reduced the abundance of the commensal *Lactobacillus.*

Holman and Chénier [147] observed that pigs which were fed tylosin-supplemented feed exhibited a rapid increase in tylosin-resistant fecal anaerobes while Rettedal et al. [148] observed that piglets which were fed chlortetracycline for two weeks following weaning did not have an increase in chlortetracycline resistance in total anaerobes but displayed a significant change in the ileal microbiota characterized by a decrease in *Lactobacillus johnsonii* and *Turicibacter* and an increase in *Lactobacillus amylovorus*. An example of the long-term effect of antibiotics on the gut microbiota was reported by Looft et al. [149], who found that in feed-antibiotic administration (chlortetracycline 100 g per ton, sulfamethazine 100 g per ton and penicillin 50 g per ton) for two weeks significantly affected the 3-months-pig gut microbiome by reducing the *Lachnobacterium* spp. and increasing the *E. coli* which contributed to an impairment of the aromatic amino acids and derivative subsystem in the gut [149]. Bosi et al. [150] found that amoxicillin, tilmicosin and doxycycline, after three weeks of continuous administration post-weaning, had a different effect on total *Lactobacillacea* and *Enterobacteriace* fecal shedding, with an increase of the ratio *Lactobacillacea* to *Enterobacteriacea* for tilmicosin and doxycycline only and a decrease of *Lactobacillacea* counts with amoxicillin, compared to the control.

On the other hand, other studies evidenced that antibiotic administration does not change the gut microbial profile of post-weaning piglets. Kalmokoff et al. [151] reported no impact on the fecal microbiota of pigs fed either tylosin or virginiamycin. Poole et al. [152] observed no effect of chlortetracycline on the fecal microbiota of post-weaned pigs which were fed it for a 28-day period. They suggested that a lack of action of the antimicrobial growth promoters on the fecal microbiota may have been due to the fact that the antimicrobials could have acted on the gastrointestinal tract prior to reaching the last part of the intestinal tract.

It is obvious that, although many studies have investigated the effect of antimicrobials on the pig intestinal microbiota, there is no consensus. Considering the same antimicrobials, differences in specific taxa can be observed; e.g., the relative abundance of phylum Bacteroidetes and the lactic acid bacterial genera *Lactobacillus* and *Streptococcus*, as well as *Coprococcus*, has been reported to be both increased and reduced following in-feed antibiotic administration according to the doses, the intestinal segment investigated and the timing of the sampling [147,149,153,154,155]. Nonetheless, the resilience of the pig gut microbiota and of the dominant taxa to long-term changes due to the administration of antimicrobials is apparent [153,156].

## 4. Conclusions and Perspectives

This review summarizes the main factors affecting the gut microbial profile of piglets in the pre- and post-weaning stages and discusses the most recent literature regarding these factors. The effects of the maturation of the microbiota with age and the effects on the diet and the body weight are clear and confirmed by various articles. Although, in the present review, the effect of diet is only been marginally treated, this factor has certainly been confirmed to be one of the main factors capable of modifying the microbial profile, and nutritionists need to shift attention from feeding the pigs/sows toward also feeding the gut microbiota. This review also points out several gaps; the effect of weaning age, the use of creep feed and the key role of the composition of colostrum and milk are among these factors. Similarly, the effect of the use of antibiotics also seems to produce controversial results. Furthermore, the role of genetic selection plays in improving the intestinal eubiosis of the pig during its early stages of growth should also be additionally investigated.

The literature analyzed does not provide a clear picture regarding the interventions which should be specifically targeted to promote the gut physiological development of pigs by modulating the gut microbiota, especially in newborns. Overall, the state of the art on gut microbiota for pigs is based on the description of taxonomical modification under specific conditions while very few data are available regarding the functional modification of the microbiota, namely as data obtained by whole genome shotgun sequencing and complete microbiome phenotyping or the connection between taxonomic data and produced metabolites. This lack of knowledge is a bottleneck to deeply understanding the intimate relationship between pig physiology and pig microbiota. Generating functional data coupled with metabolomic data is a priority for moving on to the next step of the exploitation of microbiota potential in modulating pig physiology.
